# The many facets of sustainability in medicinal chemistry: our personal experience

**DOI:** 10.1039/d5md00882d

**Published:** 2025-12-30

**Authors:** Bianca Martinengo, Eleonora Diamanti, Elisa Uliassi, Maria Laura Bolognesi

**Affiliations:** a Department of Pharmacy and Biotechnology, Alma Mater Studiorum – University of Bologna Via Belmeloro 6 40126 Bologna Italy marialaura.bolognesi@unibo.it

## Abstract

Sustainability cannot be an afterthought; it must be embedded into every framework of medicinal chemistry. This opinion piece explores how integrating the dual pillars of Green Chemistry and One Health into drug design and development may drive innovation. Through case studies using renewable feedstocks, we highlight opportunities to create medicines that are effective, ethically sound, and environmentally responsible, for a more sustainable future.

## Abstract 2

Sustainability cannot be an afterthought; it must be embedded into every framework of medicinal chemistry.

## Graphical abstract

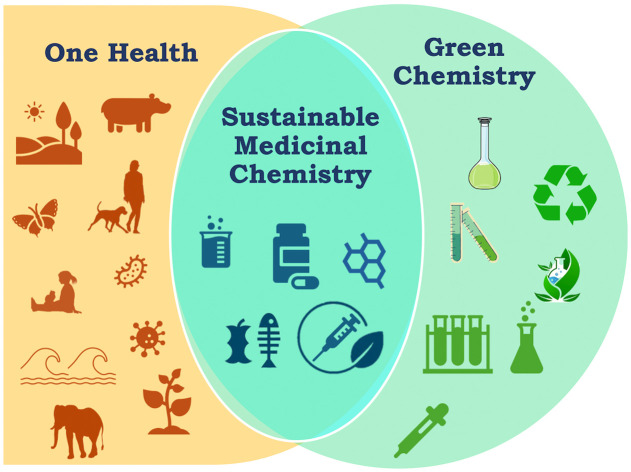

Sustainability is one of the defining challenges of our time, influencing not only our daily lives as global citizens but also shaping every field of science. In medicinal chemistry, the integration of sustainable practices has long been a topic of interest.^1^ As early as 2005, the medicinal chemistry subgroup of the American Chemical Society's Green Chemistry Institute Pharmaceutical Roundtable (ACS GCI PR)^2^ was established with the aim of providing the community with the first perspective on sustainable practices. Two decades later, the landscape has expanded considerably, now encompassing a wide range of greener processes, reagents, and enabling technologies, as well as digital tools such as electronic laboratory notebooks.^3,4^

Yet the field continues to evolve rapidly, prompting us to ask: “How many ways are there today to embed sustainability in medicinal chemistry?”.

While the principles of Green Chemistry^5,6^ provide a strong foundation and framework for integrating sustainability into medicinal chemistry, we believe these concepts can be further expanded and tailored to account for the unique considerations associated with chemicals intended to treat living organisms.

The 5th anniversary of the RSC Medicinal Chemistry dedicated to breakthroughs and future developments within the field offers a timely opportunity to reflect on these questions and to invite the broader medicinal chemistry community into the conversation. In this opinion piece, we aim to share our perspective, supported by examples from our research, to ignite discussion and further stimulate reflection on the many opportunities our field has to advance towards more sustainable drugs.^7^

The inherently complex and multifaceted nature of the drug discovery pipeline, coupled with increasing pressure to rapidly deliver potent and selective molecules, might lead to sustainability being overlooked or dismissed as a peripheral concern, or worse, as a trendy imposition. However, integrating greener and more efficient strategies from the very outset of a medicinal chemistry project can significantly influence the downstream processes and enhance the overall sustainability profile of future drug candidates.^8^

One of the many relevant aspects concerns waste minimization.^1,6^ While it is often assumed that the environmental burden lies predominantly within process development and large-scale drug manufacturing, it is important to point out that also the discovery phase itself accounts for a substantial chemical waste.^9,10^

In the early stages of a project, when the structure of a candidate molecule is still undefined, medicinal chemists were used to rely on an array of reactions that enabled the rapid generation of analogues. Working under time constraint often meant prioritizing synthetic feasibility above other considerations. In recent years, however, the shift toward sustainable alternatives have been increasingly considered and adopted with encouraging success.^8^ In this context, late-stage functionalization (LSF) strategies^11,12^ have significantly expanded the medicinal chemist's toolbox^13^ and with it, the range of accessible molecular architectures. However, they may also bring an increased demand for reagents, transition metals, solvents, and energy, and inevitably generate additional waste.

Importantly, there is growing recognition that the choices made at these early stages – about chemical routes, processes, solvents, and technologies – tend to be fixed as projects advance. Once a molecule progresses toward development or manufacturing scale-up, revising these choices might become extremely challenging or prohibitively costly. Consequently, early synthetic decisions exert a disproportionate influence on the overall environmental footprint of a drug.^9^ For this reason, industry (and academia increasingly) rely on tools^1^ that quantify sustainability metrics, minimise the use of non-preferred solvents^14^ and reagents,^4^ and recommend greener alternatives for frequently employed transformations – including photochemistry, electrochemistry, and mechanochemistry.^15^

However, in medicinal chemistry, we believe the focus on sustainability should extend beyond the adoption of eco-friendly, safe, and energy-efficient synthetic procedures. Two additional dimensions warrant equal attention: (i) the design of therapeutically effective drugs that minimise adverse impacts on human and animal health as well as the environment,^3^ and (ii) the social dimension of sustainability, namely the development of pipelines that support equitable access to medicines, by integrating economic viability, environmental impact, and social equity considerations.^16^ This is essential to improve global health and quality of life and leave no one behind.^7^

Due to their inherent biological activity, pharmaceuticals (as well as agro-chemicals and personal care products) can contribute to ecotoxicity and affect a wide range of living species.^17^ In this context, the critical question is not only how a drug interacts with its human target (or with an animal target in the case of veterinary medicines), but also how it interacts with the world around it, including the ecosystems it may ultimately impact.^18^ As medicinal chemists, we have the primary responsibility to consider the environmental fate of the molecules we design, at the very start of the drug discovery pipeline.^3^ In this regard, computational tools that now deliver robust ADME–Tox predictions – thereby improving early assessments of human health risks and reducing reliance on animal studies – could be further extended and more broadly adopted to support ecotoxicological evaluation as well.^19,20^

The second dimension of sustainability is even more complex, and research in this area continues to evolve. At Nelson Mandela University, the overarching vision is to employ the most sustainable chemistry and technologies to identify the lowest-cost manufacturing routes to active pharmaceutical ingredients (APIs).^21^ In the realm of drug discovery, Professor Kelly Chibale is Founder and Director of University of Cape Town (UCT) Holistic Drug Discovery and Development (H3D), Africa's first integrated drug discovery and development centre at the University of Cape Town, which has championed efforts toward developing affordable treatments for diseases such as tuberculosis.^21^

Here, we present examples from our own work to illustrate how sustainability might be meaningfully pursued, even within a medium-sized academic laboratory and without compromising scientific novelty or productivity. Specifically, our efforts have been guided by a simple strategy that merges two fundamental pillars of drug sustainability.^22^ The first is obviously the Green Chemistry principles, and the second is the One Health approach,^23^ which aims to sustainably balance and optimize the health of humans, animals, and ecosystems.^19^

The combination of these two key concepts has been embodied into the development of effective hit and lead molecules for vector-borne parasitic diseases (VBPD) starting from an agro-industrial waste,^24^ such as the cashew nutshell liquid (CNSL). CNSL, which is generated during processing of cashew nut, is produced globally in quantities exceeding 1 million tons each year.^24^ Its main chemical components are: anacardic acid, cardanol, cardol, and 2-methylcardol (Fig. 1). These phenolic lipids have a very peculiar chemical structure, with an alkyl side chain of 15 carbon atoms in the *meta* position with respect to their hydroxyl group(s), exhibiting varying degrees of unsaturation (Fig. 1).^25^

**Fig. 1:**
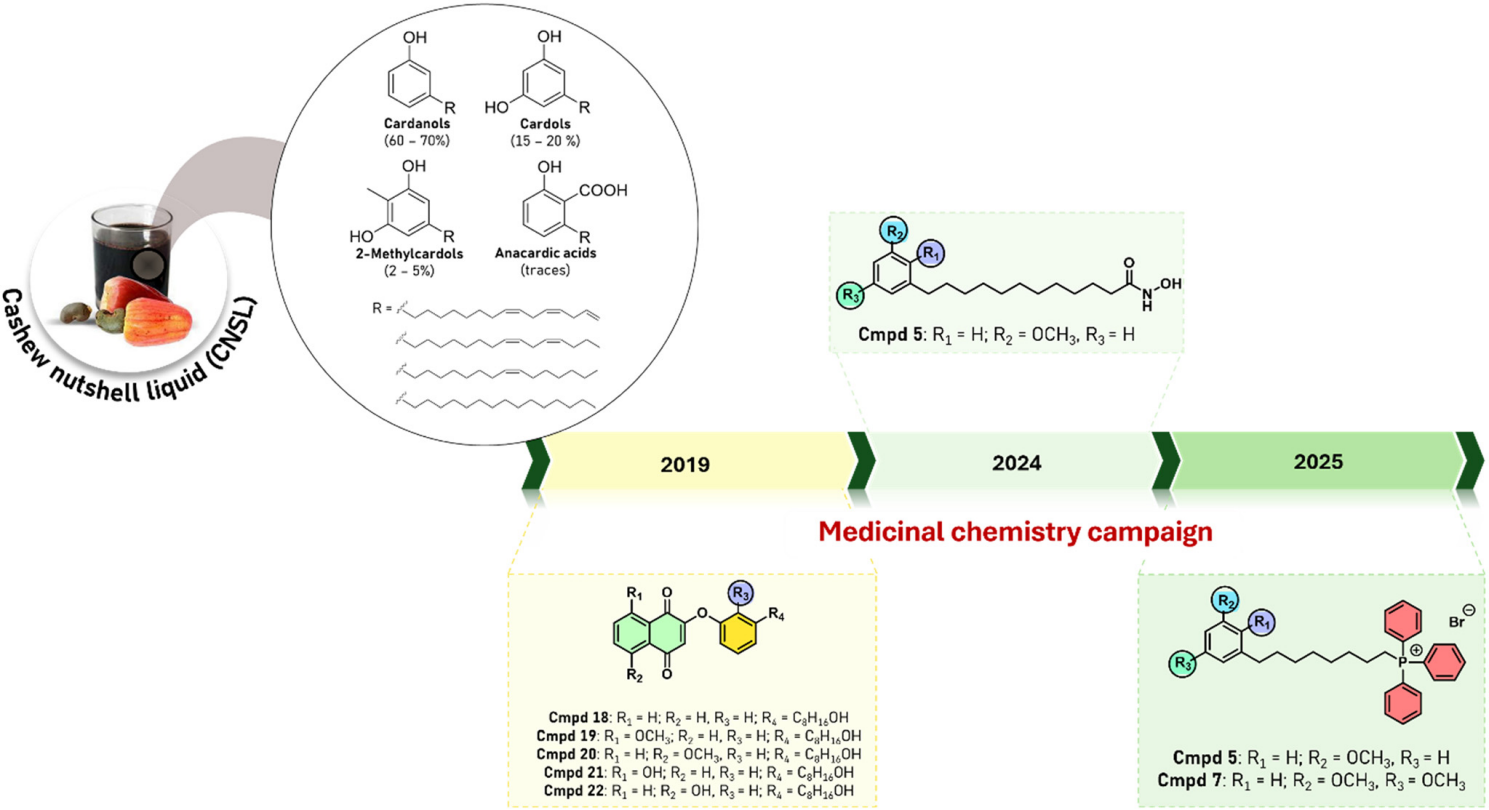
Main CNSL components and more sustainable CNSL-based derivatives with antiparasitic activity.

Although endowed with an intrinsic biological activity, CNSL components are not potent enough as drug candidates. Thus, we aimed at synthetic elaboration and diversification towards more drug-like biologically active molecules through medicinal chemistry.^26^

Importantly, being an inedible, largely available agro-industrial waste, CNSL might have environmental, financial, and ethical advantages over synthetic precursors and even natural products, in line with the Green Chemistry Principle #7 “*Use of Renewable Feedstocks*”. We were also drawn to its potential contribution to social sustainability: Africa, the Amazon basin, and the South-East Asia are among the largest cashew nut-producing countries, opening up exciting opportunities to engage endemic countries as crucial actors in VBPD drug development,^26^ by establishing local pharmaceutical industries that address public health needs, stimulating economic growth, creating jobs, reducing pharmaceutical import dependence, and minimizing carbon emissions from long-distance transportation.^7^

Building on the integration of the two pillars, our goal was to integrate sustainability concepts into the development pipeline of new classes of anti-VBPD compounds that are potentially more active against human and animal parasites than current pharmacological options, while being more sustainable in a broad sense.^26^

In 2019, our group proposed for the first time to combine CNSL derivatives with a quinone scaffold, which is known for its anti-trypanosomal activity (Fig. 1). We developed hybrid molecules that exhibited antiparasitic activity against *Trypanosoma brucei brucei* (*T. b. brucei*), the causative agent of African animal trypanosomiasis, without exhibiting human cytotoxicity.^27^ Because this is a devastating parasitic disease that affects domestic animals in many countries in sub-Saharan Africa and a huge problem to Africa development, these molecules are exciting hits for One Health solutions. In addition, as they derive from a food waste, they represent – as far as we know – the first example of sustainable-by-design hit identification, in line with Green Chemistry Principle #7.

To make our medicinal chemistry strategy greener, we developed a second library of CNSL-based antiparasitic compounds obtained through a green metathesis approach (Fig. 1). By combining two “Green Chemistry” principles (*i.e.*, #7-use of renewable feedstock and #9-catalysis), we have been able to develop derivative 5 as an interesting hit compound toward *T*. *b*. *brucei* synthesized thorough an efficient ruthenium-catalyzed cross-metathesis reaction. Recently, we have refined our medicinal chemistry strategy aimed to enhance antiparasitic activity of CNSL. We pursued a ligand-based approach that minimizes synthetic complexity and the number of reaction steps. In detailed, we have developed *via* a three-step synthesis CNSL-derived phosphonium salts where C8 alkyl chains have been combined with lipophilic cations. Strikingly, these novel compounds showed sub-nanomolar activity against veterinary trypanosomes, including *T. b. evansi* and *T. b. equiperdum*. Compounds 5 and 77 outperformed reference drugs, showing high selectivity indices (>1000) and no cross-resistance with current therapies, consistent with a mitochondrial mode of action. As a further step, we considered the compounds' ecotoxicological profile, first by predicting it (ECOSAR v2.2)^28^ and by testing it against aquatic species. Notably, 5 and 7 demonstrated lower (eco)toxicity than antiparasitic activity, highlighting their potential as environmentally safer, sustainable agents fully aligned with One Health and Green Chemistry principles.^29^ Nevertheless, poorly degradable compounds, which can persist and accumulate in soil, water, and other compartments, may lead to long-term exposure and potential adverse effects on non-target organisms, even at low environmental concentrations. This emphasizes again the importance of considering persistence, bioaccumulation, and long-term exposure risks in the environmental assessment at the early stage.^17^

Furthermore, to avoid trivializing the topic, we would like to remark that starting from a waste material, although adherent to the principle #7 of Green Chemistry, is not going to solve all the problems. Among others, there are two critical points to be considered: (i) our molecules are just hit compounds, typically requiring substantial optimization before evolving into APIs. Thus, it remains speculative whether such optimized compounds would still be accessible from the same waste sources; (ii) while using waste-derived materials is indeed sustainable in principle, the energy and solvent demands required to isolate these starting materials should not be overlooked. In the specific case of CNSL, industrial production relies on extraction processes that may involve significant resource consumption, including solvent-intensive approaches (*e.g.*, Soxhlet extraction using hexanes, petroleum ether, and other fossil-derived solvents), and the separation of its components requires additional solvent-heavy processing. Thus, the extent to which the use of CNSL offers environmental advantages depends on the specific industrial production method employed.

As we stand at a crossroads in the evolution of medicinal chemistry, we have the chance to influence the future of drug discovery, ensuring that both efficacy and sustainability are prioritised. The path forward is clear: sustainability is not just an ancillary concern; it is a fundamental element of drug development that is going to define the entire pipeline in the years to come. As scientists and innovators, medicinal chemists have the responsibility and the opportunity to lead the way in creating a diverse set of more sustainable, ethically sound, and impactful approaches to drug design and development. The time to act is now, and the future of medicinal chemistry depends on the decisions we make today. Sustainability should not be seen as a constraint or an added burden on an already demanding and complex field. Instead, the vision of developing new drugs that embrace sustainability, both environmentally and ethically, can and should become a reality.

## Conflicts of interest

There is no conflict of interest to declare.

## Data Availability

No primary research results, software or code have been included, and no new data were generated or analysed as part of this review.
